# EU-OPENSCREEN—chemical tools for the study of plant biology and resistance mechanisms

**DOI:** 10.1007/s12154-014-0118-9

**Published:** 2014-07-31

**Authors:** Torsten Meiners, Bahne Stechmann, Ronald Frank

**Affiliations:** 1 Leibniz-Institut fuer Molekulare Pharmakologie (FMP), Robert-Roessle-Strasse 10, Berlin, 13125 Germany; 2 Helmholtz-Centre for Infection Research (HZI), Inhoffenstrasse 7, Braunschweig, 38124 Germany

**Keywords:** High-capacity screening, European research infrastructure, Tool development, Compound collection, Open access, Biological assays

## Abstract

EU-OPENSCREEN is an academic research infrastructure initiative in Europe for enabling researchers in all life sciences to take advantage of chemical biology approaches to their projects. In a collaborative effort of national networks in 16 European countries, EU-OPENSCREEN will develop novel chemical compounds with external users to address questions in, among other fields, systems and network biology (directed and selective perturbation of signalling pathways), structural biology (compound-target interactions at atomic resolution), pharmacology (early drug discovery and toxicology) and plant biology (response of wild or crop plants to environmental and agricultural substances). EU-OPENSCREEN supports all stages of a tool development project, including assay adaptation, high-throughput screening and chemical optimisation of the ‘hit’ compounds. All tool compounds and data will be made available to the scientific community. EU-OPENSCREEN integrates high-capacity screening platforms throughout Europe, which share a rationally selected compound collection comprising up to 300,000 (commercial and proprietary compounds collected from European chemists). By testing systematically this chemical collection in hundreds of assays originating from very different biological themes, the screening process generates enormous amounts of information about the biological activities of the substances and thereby steadily enriches our understanding of how and where they act.

## Introduction

Small molecules have many advantages over proteins or nucleic acid-based agents for studying biological systems; for instance, they are chemically well defined and can be provided in reproducible quality; small molecules can also be chosen to be cell-permeable and selectively modulate intracellular processes in an instant and reversible competitive fashion with their cellular targets. Many fundamental questions about the molecular mechanisms which underlie biological processes can be addressed advantageously—and sometimes exclusively—by using chemical tools. Over the past decades, gene technology approaches have brought about multiple ways in which to manipulate biological systems. However, although numerous genetic resources and tools are available, many biological processes and pathways cannot be addressed appropriately via genetic means due to gene redundancy and/or embryonic lethality. Small molecules can overcome these limitations because their application can be readily dosed and controlled in a temporal and spatial manner.

Principles of biological processes hold true across all living species, be they humans, animals, bacteria, fungi, worms, plants, fish, algae, viruses etc. The search for novel chemical tools is thus very similar and a major access route is to develop an assay specific for the target under study and to use a facility where the assay can be screened against a collection of compounds to find an active substance, or a ‘hit’. After this, a chemist will often need to improve the properties of that hit to make it a useful tool compound.

Currently, large-scale chemical biology initiatives almost exclusively focus on human disease mechanisms, while the application of the small molecule approach in other life sciences is lagging. Therefore, EU-OPENSCREEN, a pan-European infrastructure initiative to support academic research in chemical biology, expressly advocates for the wider use of chemical tool compounds and will serve projects from disciplines in which scientists have not yet tapped the full potential of chemical biology, such as plant biology, marine and microbial biology and ecology.

The results of this chemical biology research will primarily provide a deeper knowledge of how biological processes and which cellular targets can be selectively influenced and the consequences of this for entire cells, species and ecological systems. Understanding how chemicals, both natural and artificial substances, affect our lives and environment, is also of critical importance for the development of new and safer products—be they drugs to treat diseases, herbicides to protect crops or food additives for livestock. As such, both industry and the wider society share a great interest in chemical biology. Any chemical released into the biosphere will, in some way, interact with living species, affecting them in ways both beneficial and potentially adverse; a detailed knowledge about how chemicals act is vital for society as well as its economy, and for patients, consumers and industry.

**Fig. 1:**
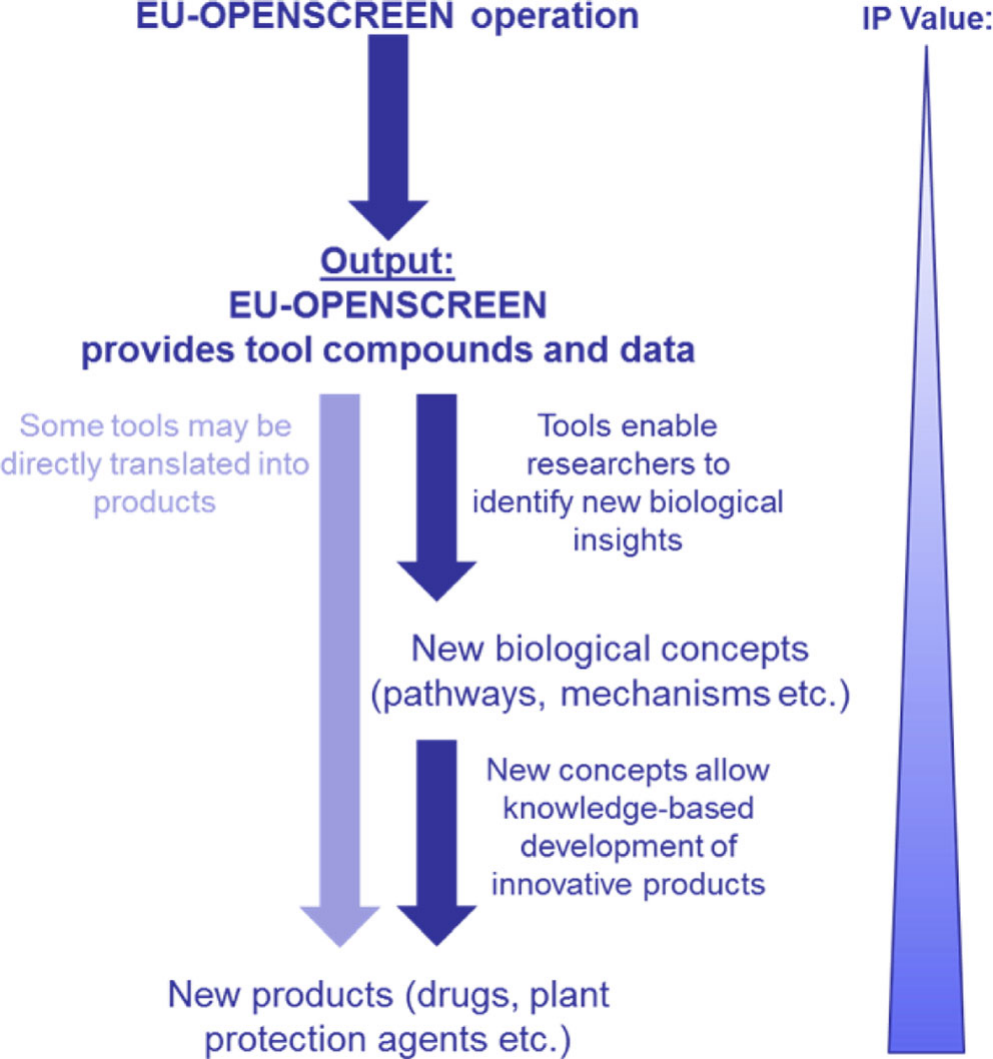
Research output and impact on innovation at the EU-OPENSCREEN infrastructure

EU-OPENSCREEN will promote innovation in two ways (Fig. 1). First, tool compounds themselves can be starting points for product development and directly translated into commercial application. Secondly, tools validate their targets/processes to be ‘druggable’ which is an attractive situation for the knowledge-driven development of innovative products based on target function. The intellectual property (IP) value of the research data significantly increases during later development stages, with EU-OPENSCREEN forming the basis for generating valuable IP and innovation.

## Need for a chemical biology infrastructure

Modern DNA sequencing technologies can rapidly read the genomes of man or other species and unravel thousands of novel genes within days, but the functions of these genes (and their roles in diseases, for instance) remain largely unknown. The availability of suitable ‘tools’ for systematic biochemical investigation of their function is severely lagging.

One major route for the discovery of bioactive substances is the systematic empirical screening of large compound collections with dedicated bioassays that have been designed to respond with a robust signal to an anticipated biological activity. This approach, commonly applied by industry, is technically and logistically demanding and requires large, dedicated facilities with expensive instrumentation and experienced personnel which are unavailable to many researchers. Several academic institutions have recently established such high-tech screening facilities in Europe to support interdisciplinary research projects between chemists and biologists. It is the objective of EU-OPENSCREEN to integrate these facilities within a truly pan-European structure, similar to the NIH’s Molecular Libraries Probe Production Centers Network (MLPCN) [2]. This would generate the critical mass to cost-effectively develop novel tools to the benefit of the scientific community and society. A pan-European infrastructure to support academic research in chemical biology, with the name of EU-OPENSCREEN, is currently under development.

## European strategy forum on research infrastructures (ESFRI)

**Fig. 2:**
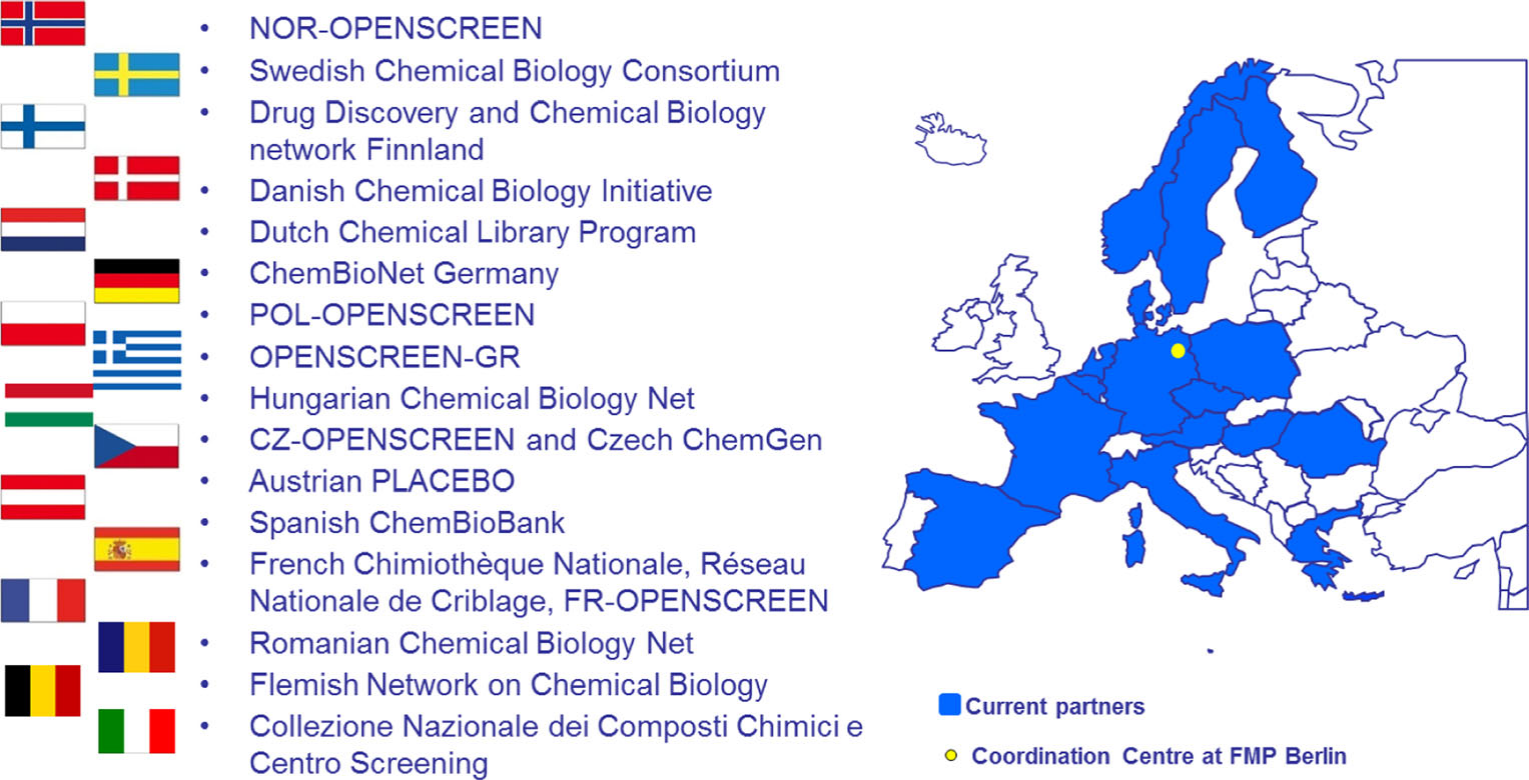
EU-OPENSCREEN national networks for chemical biology

Established in 2002 by the EU Competitiveness Council and the research ministers of the European member states, ESFRI developed a European Strategic Roadmap for Research Infrastructures that are ‘vital to the excellence of research and innovation in Europe’. With the inclusion of EU-OPENSCREEN on the roadmap, ESFRI grants chemical biology a high priority along with 12 other research fields in the thematic area of biological and medical sciences, ranging from bioinformatics, structural biology and bio-banking to ecosystems, marine and microbial diversity (European Strategy Forum on [10]). EU-OPENSCREEN, currently in its preparatory phase and funded by the European Commission, builds on national networks in 16 European partner countries and their expert facilities with many years of proven high-quality services (Fig. 2). The funding for the construction and operation of this research infrastructure will mainly be provided by individual member states. As of July 2014, ten countries and the EMBL-EBI have expressed their interest in participating in EU-OPENSCREEN by signing a memorandum of understanding. EU-OPENSCREEN plans to create a European Research Infrastructure Consortium (ERIC) legal entity.

## Chemical biology infrastructure concept

**Fig. 3:**
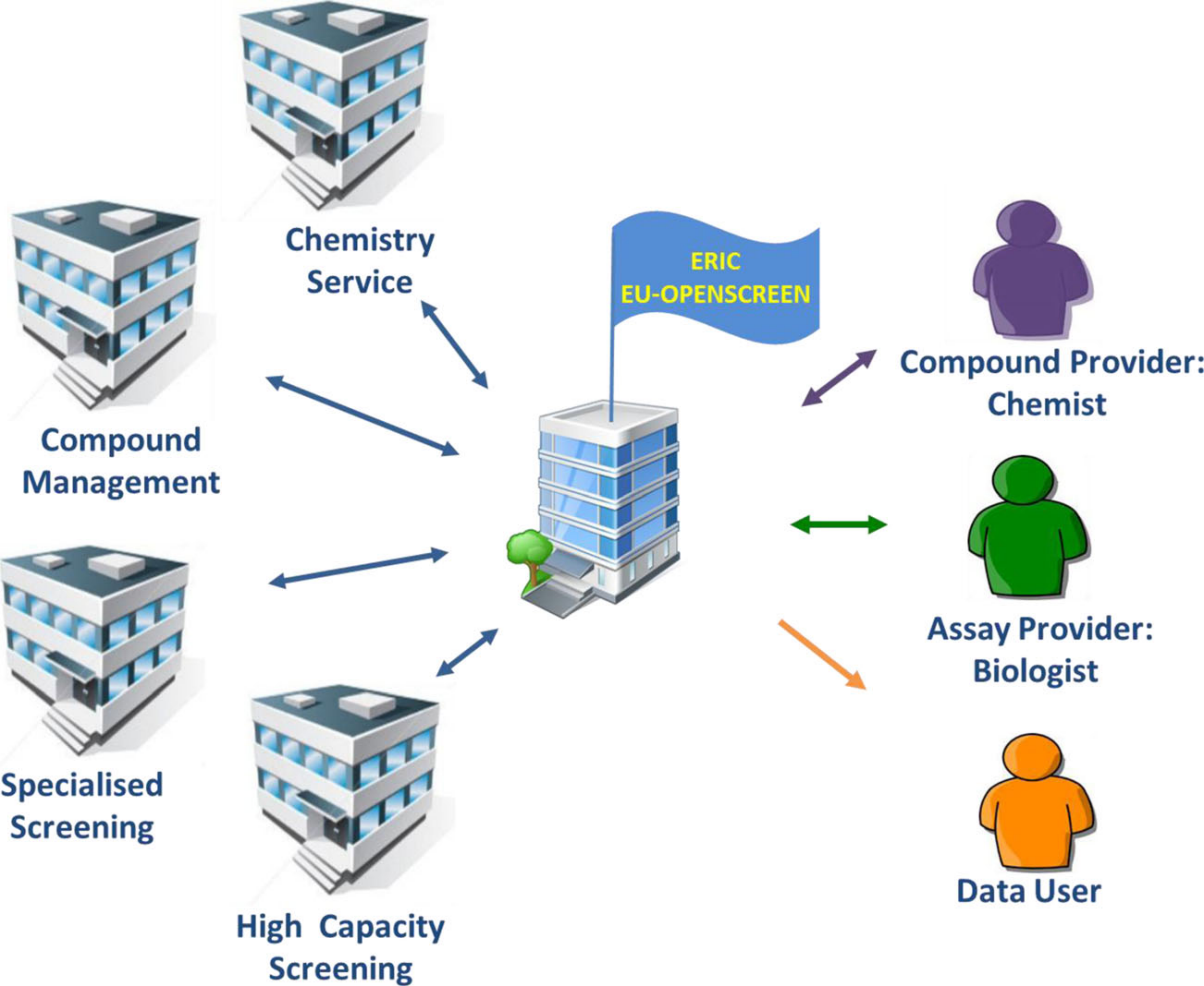
Organisation scheme of the distributed research infrastructure EU-OPENSCREEN (see text for explanations)

EU-OPENSCREEN is dedicated to accelerating the discovery of biologically active substances in all areas of life sciences and has developed a process and structure for its service activities involving a multitude of research centres and a wide range of technologies and expertise, in order to optimally serve the broader biological community. The concept is to: (i) build a central joint chemical compound collection (European Chemical Biology Library, ECBL) that draws on an abundant and relevant chemical diversity to form a unique resource, (ii) distribute this collection to the European partner facilities which are experts in their distinct themes and embedded in excellent research centres, (iii) support outstanding research projects by channelling these through a central hub to the most appropriate facilities in Europe and (iv) unite all information generated from the projects in a central database (European Chemical Biology Database, ECBD) and make data and tools available to the public (Fig. 3). The compound collection will be managed (storage, QC, distribution of compounds to the service sites) in a central compound collection management facility. Partner institutes will adhere to the best operational standards so as to enable cross-experiment data analyses. A central office manages the operation of the research infrastructure and the user access, and serves as the single contact point for users.

All compounds entering the compound collection will be quality controlled by established analytical methods (liquid chromatography–mass spectrometry, LC-MS) and characterized for a standard set of properties (bioprofiling); around 40 data points will be measured for each compound covering chemical, biophysical, cellular cytotoxic, antimicrobial and other activities. Chemists donating their compounds will instantly receive this information on the property profiles of their substances as a compensation for their donations.

Producing and handling libraries of chemicals involves dealing with a high degree of complexity not only during collection, validation and curation of the libraries, but also when generating the chemical complexity with the chemical space of the collection. Chemical diversity is a widely applied concept to select structurally diverse subsets of molecules with the aim of maximizing the number of hits in biological screening [8]. While many methods exist in the area, few systematic comparisons using current descriptors in particular with the objective of assessing diversity in bioactivity space have been published. Koutsoukas et al. [8] compared 13 widely used molecular descriptors and assessed both the similar behaviour of the descriptors in assessing the diversity of chemical libraries, and their ability to select compounds from libraries that are diverse in bioactivity space, which is a property of much practical relevance in screening library design.

The ECBL will be composed with respect to maximal diversity/coverage of chemical space, aiming at providing hits of a large spectrum of druggable targets. The conception, sources and characteristics of the ECBL are detailed in [7]. Here, it is explained how the selection task of 200,000 molecules out of a pre-filtered set of 1.4 million candidates was shared by five independent European research groups from the EU-OPENSCREEN consortium, each picking a subset of 40,000 compounds according to their own in-house methodology and expertise. The descriptor vectors used had included binary fingerprints, substructure/pharmacophore pattern counts or descriptors of predicted molecular properties, implicitly substituting the typical CS by a property space in order to maximize the number of represented molecular scaffolds/substructures [7]. The compound selections contributed by the various participating groups were mapped onto general-purpose self-organizing maps built on the basis of marketed drugs and bioactive reference molecules. In this way, the occupancy of chemical space by the EU-OPENSCREEN library could be directly compared to distributions of known bio-actives of various classes [7].

Focusing its efforts with those of other continents EU-OPENSCREEN has started a cooperation with Compounds Australia— Australia’s national collection of substances [11] to provide European researchers with access to compounds formulated by Australian chemists and vice versa.

EU-OPENSCREEN will implement an open-access policy to encourage maximal data dissemination and publication, where the same rules will apply to users from academia and industry alike. An optional 18-month embargo period to withhold the data is permitted in order to allow for translation of research results. Together with sibling initiatives of the biological and medical infrastructures on the ESFRI roadmap, EU-OPENSCREEN has developed a position paper on the principles of data management and sharing among European Research Infrastructures [3].

EU-OPENSCREEN’s database—the European Chemical Biology Database (ECBD), hosted at the European Bioinformatics Institute in Hinxton, UK, will contain validated output from the screening centres in a public and pre-release form, offering citable links back to the originator laboratories for primary raw data. It offers a web portal (https://www.eu-openscreen-data.eu/) with search and analysis capabilities and is designed to ensure the maximal availability, reuse and analysis of data. One of the most central standards that will be adopted by ECBD is the use of the standard International Chemical Identifier (InChI). This has rapidly become the worldwide de facto standard for organic small-molecule structure representation and allows for compounds to be simply normalised within the ECBD, and then, for these representations to be straightforwardly integrated with other chemical resources on the internet such as for structure–activity relationship (SAR) studies (ChEMBL), chemical structure (PubChem) and target (UniProt) resources. ChEMBL is an open data database containing binding, functional and ADMET information (from >5 million bioactivity measurements and >5,000 protein targets) for a large number (>1 million) of drug-like bioactive compounds (see [6] for a detailed description). It can be accessed through a web-based interface, data downloads and web services at: https://www.ebi.ac.uk/chembldb.

**Fig. 4:**
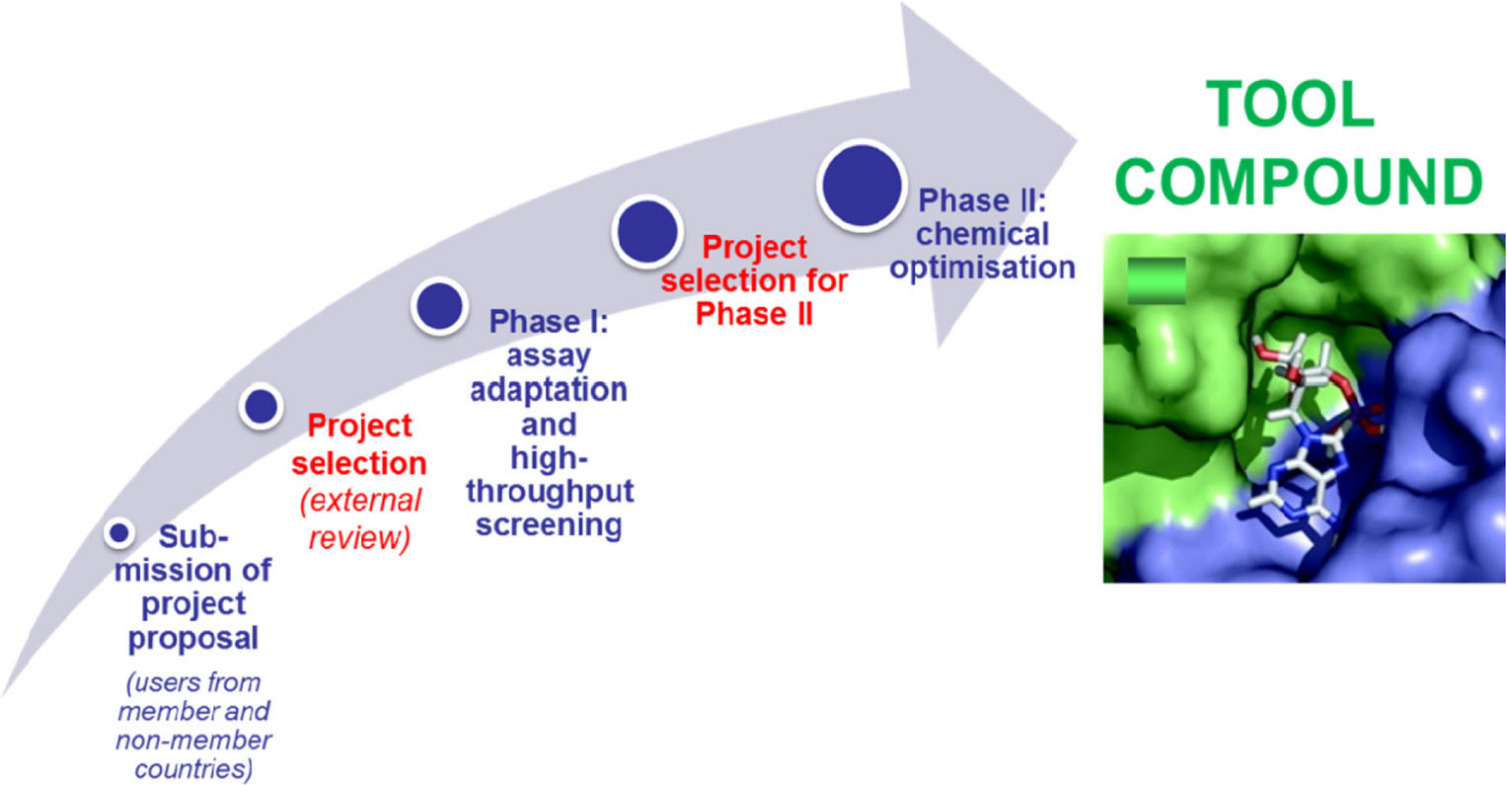
EU-OPENSCREEN will offer services, resources and expertise which support all stages of a tool development project. Its core activity is the systematic screening of large diverse compound libraries (phase I) and the chemical optimisation of the identified compounds into valuable tool compounds (phase II)

Tool development projects will be selected according to scientific novelty and excellence. EU-OPENSCREEN will support these projects through assay development, screening and follow-up chemical optimisation as well through as biological validation (Fig. 4) and will thus make high-quality tool compounds available to the scientific community.

## EU-OPENSCREEN and the resistance challenge in agriculture

In plant sciences, chemical biology represents a powerful tool for studying plant biology. Plant chemical biology is the application of bioactive small molecules to study cellular networks and developmental processes in plants. In recent years, plant scientists have successfully used ‘chemical tools’ to address fundamental questions about plant biology, such as intracellular transport processes, cell wall biology, and plant hormone signalling (reviewed in [1]). These synthetic molecules have the potential to be developed as agrochemical leads, while vice versa, the research on agrochemicals has generated plant chemical biology knowledge and tools for basic research [12].

The EU-OPENSCREEN infrastructure for chemical biology can help to address the resistance challenge in agriculture by generating chemical tools for understanding resistance mechanisms and by identifying new modes of action unaffected by resistance. It can thus help to satisfy the need for effective research into new crop-protection compounds by seeking novel active ingredients and by improving the identification process of new targets. All of this is necessary because losses in agricultural yields caused by insect damage, fungal pathogens and competition by weeds is in the range of 13–15 % for each of these three detrimental agents and new chemical tools are needed to combat them and reduce yield losses [4]. The urgency of this problem is illustrated by the fact that no chemistries with a new mode of action have been introduced as herbicides in the last 30 years [5]. Moreover, resistance of agricultural weeds and pests to chemical control is a problem of growing importance and serious consequences. Cutting-edge developments in the design and synthesis of agrochemicals help to tackle today’s challenges of weed and pest resistance [9].

An alternative crop-protection strategy is the application of chemicals that exert their biological effects not in the pathogen, but rather directly in the plant treated, e.g. by mimicking plant hormones and priming plant defence responses to elicit protection against fungal and bacterial pathogens or insect pests. Besides crop-protective substances, chemical compounds can also be used as plant growth regulators for plants and yield improvement by inducing favourable phenotypes [12]. One example is compounds that interfere with ethylene responses and have a useful application in the post-harvest quality of fruits and vegetables [13].

Taking into consideration the recent contributions of small molecules to plant biology and agrochemical products, it is evident that access to novel and unique chemistries and connecting these to physiologically relevant processes in plant assay systems will open up new avenues to novel chemical tools tailored to plant biology and potential agrochemical applications. While the agrochemical industry has built up and successfully used modern agrochemical research platforms for high-throughput screening (HTS) during the last 15 years [4], EU-OPENSCREEN offers academia and small and medium enterprises its infrastructure as a platform and attractive option for HTS. The hundreds of screens performed at EU-OPENSCREEN will generate broad knowledge on the bioactivities of chemicals and the responses of biological systems (cells, tissue, plants, invertebrate and vertebrate animals) upon challenge with these chemicals. This knowledge is accessible in the ECBD (https://www.eu-openscreen-data.eu) and can be used to predict the eco-toxicity of potential leads. EU-OPENSCREEN will use the significant expertise available at specialised partner screening sites to support users from the field of plant chemical biology when it starts operating as a permanent European Research Infrastructure Consortium in 2016. First calls for projects will be released end of 2015.
